# Fruit and vegetable consumption and depression symptoms in young women: results from 1973 to 1978 cohort of the Australian Longitudinal Study on Women’s Health

**DOI:** 10.1007/s00394-022-02926-8

**Published:** 2022-07-21

**Authors:** Putu Novi Arfirsta Dharmayani, Gita D. Mishra, Seema Mihrshahi

**Affiliations:** 1 Department of Health Sciences, Faculty of Medicine, Health and Human Sciences, Macquarie University, Sydney, NSW 2109 Australia; 2 School of Public Health, University of Queensland, Brisbane, QLD 4006 Australia

**Keywords:** Fruit, Vegetables, Depression symptoms, Young women, Nutrition, Diet

## Abstract

**Purpose:**

Growing evidence suggests that specific food groups may play an important role in improving mental health. However, very few studies explored the association between individual dietary factors and depression symptoms by following a large cohort of individuals over a long period. We examined the differential effects of fruit and vegetables in relation to depression symptoms over a 15-year follow-up period in the 1973–78 cohort of the Australian Longitudinal Study on Women’s Health.

**Methods:**

Fruit and vegetable consumption was assessed using short questions. The Center for Epidemiologic Studies Depression-10 scale with a cut off ≥ 10 indicated depressive symptoms. Multiple imputations with generalised estimating equations models were performed to estimate odds ratio of depression symptoms according to fruit and vegetable consumption.

**Results:**

A total of 4241 participants with a mean age of 27.6 (SD 1.45) years at baseline were followed up at five surveys (2003–2018). Fruit and vegetable intake (≥ 2 servings) was cross-sectionally associated with lower odds of depressive symptoms. In longitudinal analysis, a higher intake of fruit (≥ 4 servings) and vegetable (≥ 5 servings) was consistently associated with lower odds of depressive symptoms, with a 25% lower odds (OR 0.75; 95% CI 0.57, 0.97; *p* = 0.031) and a 19% lower odds (OR 0.81; 95% CI 0.70, 0.94; *p* = 0.007) than consuming one serve or less fruit and vegetable, respectively.

**Conclusion:**

These results suggest that a higher intake of fruit and vegetables was associated with a lower risk of depression symptoms over 15 years from a population-based prospective study of Australian women.

**Supplementary Information:**

The online version contains supplementary material available at 10.1007/s00394-022-02926-8.

## Introduction

Depression is a highly recurrent, debilitating, and prevalent mental disorder in the general population that can significantly hamper adaptive functioning [1]. With the number of individuals with depressive disorder steadily rising in most countries, it is predicted that major depressive disorder (MDD) will be the leading cause of disability and disease burden worldwide by 2030 [2, 3]. According to recent findings from the Global Burden of Disease study, depression was the top-ranked cause of disability-adjusted life-years (DALYs) in the younger population [4]. A study by Rohde et al. [5] showed that emerging adulthood has the highest prevalence of MDD compared to other age groups.

A number of epidemiological studies concluded that the lifetime prevalence of depression in women is two-fold more prevalent than in men [6–8]. A meta-analysis representing data from 90 different nations showed a medium effect size of the gender difference in depressive symptoms and women having higher odds of depression symptoms compared with men [9]. There is a substantial economic burden related to health system spending for common mental disorders in several developed countries [10–12]. A recent systematic review and meta-analysis found considerably higher excess depression costs for total direct and indirect costs, notably in young people [13]. Thus, population-based prevention strategies are highly needed to reduce the incidence of depression and its economic burden.

A growing body of research suggests the potential influence of dietary patterns on common mental disorders. The role of diet in relation to health-related quality of life has been extensively investigated, with more positive associations observed with adherence to healthy dietary patterns and the Mediterranean diet [14, 15]. Evidence from at least three systematic reviews found that both dietary patterns have also been associated with a greater reduce the risk of developing depression [16–18]. A common characteristic of both dietary patterns is higher intakes of fruit and vegetables. A meta-analysis of randomised controlled trials suggests greater benefits from dietary interventions by adhering to high-fibre and nutrient-dense alternatives, such as vegetables, to reduce depression symptoms among women samples [19]. Thus, the relationship between fruit and vegetable consumption and depression symptoms is an emerging area of research.

However, several systematic reviews found conflicting findings across age groups from observational evidence [20–24]. A differential effect of fruit and vegetables was reported in studies that examined the effects of fruit and vegetables separately on depression symptoms [25–27]. Our previous results from the Australian Longitudinal Study on Women’s Health (ALSWH) study, for example, showed that greater consumption of fruit was associated with a reduced prevalence and incidence of depressive symptoms in mid-age women, whereas the effects of vegetables remained unclear [28].

Despite accumulating evidence, the associations of fruit and vegetable and depression symptoms remain relatively underexplored in young adults. Early adulthood is a period associated with low diet quality, and young adults are likely to be more susceptible to common mental disorders due to life transitions [24]. Evidence from longitudinal studies on fruit and vegetable consumption for preventing depression symptoms among young women is scarce. To address this gap, we investigate the association between fruit and vegetable consumption and depression symptoms in young women. Our study aims to examine the differential effects of fruit and vegetables in relation to depression symptoms over a 15-year follow-up in young women from the 1973–78 cohort of the ALSWH.

## Methodology

### Study sample and participants

The ALSWH is an ongoing prospective cohort study that aims to examine the associations between biological, psychological, social, and lifestyle factors and women’s physical health and emotional well-being [29]. This study has collected health data from over 40,000 Australian women from three age cohorts, born in 1921–26, 1946–1951, and 1973–78 since 1996 [30]. Women were randomly recruited from the Medicare database in 1996, which covers all Australian citizens and permanent residents. In 2012–2013, the fourth cohort born in 1989–95 was included in the study. At baseline, participants have been shown to be broadly representative of Australian women in the same age group. Further information on the ALSWH is available on the official website (https://www.alswh.org.au/).

The sample for investigating the association between fruit and vegetable consumption and depression symptoms is drawn from the 1973–78 cohort. During the initial survey, a total of 14,247 women aged 18–23 completed the survey via mail. Self-administered questionnaires were sent to participants at approximately 3-year intervals until 2018 (survey 8). Details of study design, cohort, and recruitment procedures have been described elsewhere [29]. This secondary analysis focuses on dietary data collected in the five survey waves from survey 3 in 2003 (age 25–30) until survey 8 in 2018 (age 40–45), where dietary intake short questions were administered in the questionnaires starting in 2003. Survey 3 was used as the baseline for the current study, and its response rate was 66.3% (*n* = 9081). Survey 6 was excluded from analysis because dietary data was not collected.

### Assessment of dietary intake

The daily intake of fruit and vegetable was assessed using the short questionnaires (Supplementary Table 1) [31]. Most short questions were asked the participants to report their daily intakes in serving size. A serve of fruit was equivalent to one medium piece or two small pieces of fruit, or a half cup of diced fruit, berries, or grapes, whereas a serve of vegetables was equivalent to half a cup (75 g) of cooked green or orange vegetables, or cooked dried or canned legumes, or sweet corn, or one cup of green leafy or raw salad vegetables [32]. Participants marked one only answer for each question according to their usual eating habits over the past 12 months.

Dietary intake data was collected using a food frequency questionnaire in Survey 3 and Survey 5, based on the validated Dietary Questionnaire for Epidemiological Studies Version 2 developed by the Cancer Council Victoria [31, 33]. The questionnaire was previously validated in the young to middle-aged women population [33]. Total energy and nutrient intakes were computed using Australian nutrient composition data from the NUTTAB 1995 [34]. The current study used the latest dietary intake data at survey 5 to calculate total energy intakes and fish consumption. Due to a skewed distribution, women were categorised into three groups based on their total fish consumption, namely never consuming fish, < 30 g/day, and ≥ 30 g/day, for analysis. Several systematic reviews suggested that fish or omega-3 fatty acid intake may protect against depression, particularly among women.

### Assessment of depression symptoms

The 10-item Center for Epidemiologic Studies Depression (CESD-10) scale was used to assess the presence of symptoms associated with depression over the previous week. This instrument is a shortened version of the CESD-20 scale, which was constructed and designed to measure depressive symptoms in the general adult population aged 18 or older [35]. Validation of CESD-10 against the CESD-20 using a cut-off of 10 or more has been demonstrated to minimise false-positive results with a slight loss of sensitivity [36]. The CESD-10 contains a 10-item Likert scale questionnaire, and items are rated on a four-point Likert scale ranging from 0 (‘rarely or none of the time’) to 3 (‘most or all of the time’). Scores range from 0 to 30, with higher scores indicating greater severity of depression symptoms. A cut-off ≥ 10 was used to indicate women having depression symptoms. Depression symptoms were treated as a binary (dichotomous) outcome variable in the analyses.

### Assessment of covariates

A range of potential confounding variables was considered, and information on these was obtained from each survey. In addition, a number of sociodemographic, health behaviours, chronic diseases, and history of depression symptoms were included in the modelling of the association between fruit and vegetable consumption and depression symptoms.

#### Sociodemographic variables

Sociodemographic variables included the area of residence (major cities; inner regional; outer regional/remote/very remote), ability to manage available income (no difficulty/not too bad; difficult sometimes; very difficult/impossible), the highest level of education (high school or less; trade/college; university), marital status (married/de facto; single; separated/divorced/widowed), and having a child (no; yes). These variables were reported in each survey.

#### Health behaviours

##### Smoking status and alcohol consumption

Smoking status was categorised based on the Australian Institute of Health and Welfare [37] as a non-smoker, ex-smoker, irregular smoker, weekly smoker, and daily smoker. Alcohol consumption was classified according to National Health and Medical Research Council [38] criteria (non-drinker, rarely drinks, low-risk drinker, risky drinker, and high-risk drinker). As very few women were classified as ‘high-risk drinkers’, they were combined with the ‘risky drinker’ category.

##### Physical activity

Physical activity was self-reported in each survey. Participants reported the frequency and duration of physical activities, such as walking briskly, moderate leisure activity, vigorous leisure activity, and vigorous household or garden chores, in the last week. Based on self-reported information, the amount of each activity was calculated using minutes of metabolic equivalents of task (MET-min) per week and physical activity was then categorised as ‘nil/sedentary’ (< 40 MET-min/week), ‘low’ (40 ≤ MET < 600 min/week), ‘moderate’ (600 ≤ MET < 1200 min/week), and ‘high’ (≥ 1200 MET-min/week) [39, 40].

#### Body Mass Index

Body Mass Index (BMI) was calculated as self-reported weight (kg) divided by the square of self-reported height (m) and categorised as ‘acceptable weight’ (18.5 ≤ BMI < 25), ‘underweight’ (BMI < 18.5), ‘overweight’ (25 ≤ BMI < 30), and ‘obese’ (BMI ≥ 30).

#### History of chronic disease

Participants self-reported a range of medical conditions in each survey. In the current study, individuals who provided a positive response to the short questions “in the last 3 years, have you been diagnosed or treated for: …” with any of the following conditions were considered to have a chronic disease: heart disease, hypertension, and diabetes.

#### History of depression

Depression symptoms were self-reported and participants were determined to have a history of depression if they met any of the following criteria: (a) provided a positive response on the following question in survey 2 “Have you ever been told by a doctor that you have depression in the last 4 years?” or (b) reported having the CESD-10 scores which equal to 10 or more at survey 2 or 3.

### Statistical analysis

The present analysis included participants who had complete data on fruit and vegetable consumption and the CESD-10 score across the surveys (*n* = 4241). Baseline characteristics were described by calculating their frequencies as percentages for binary and categorical variables, and means with standard deviation (SD) for continuous variables. Characteristics of participants at baseline were compared using the Chi-square test for binary and categorical covariates, and *t *test for normally distributed continuous covariates.

The primary analysis used multiple imputations in conjunction with generalised estimating equations (GEE) models to examine the associations between fruit and vegetable consumption and depression symptoms. Multivariate imputation by chained equations (MICE) was performed to handle missing data in confounders [41, 42]. Sampling variability from the imputation process can be reduced by increasing the number of imputed datasets (at least 20) [43]. Therefore, MICE was used to impute missing data on covariates with *m* = 20 chains run for 20 iterations, with a logistic regression model for a binary variable, an ordered logistic regression for ordinal variables, and multinominal logistic regression for a nominal variable. The GEE regression models estimate the average effects over 15 years, accounting for within-participants correlation at all five surveys. The GEE model can be used for cross-sectional and longitudinal analysis.

The total of the CESD-10 scores was dichotomised using the cut-off ≥ 10 to determine whether depression symptoms were present or not (0 = no depression symptoms, 1 = depression symptoms were present). To measure the daily servings, fruits were measured categorically, with three categories: ≤ 1 serve/day; 2–3 serves/day; ≥ 4 serves/day. Similarly, vegetables were classified into three categories: ≤ 1 serve/day; 2–4 serves/day; ≥ 5 serves/day. Models were adjusted for time-dependent variables reported at each survey, including area, ability to manage available income, marital status, having a child, smoking status, alcohol consumption, physical activity, BMI, and history of chronic diseases. The highest level of education in survey 8 was also added to the models. Adjustment for total energy intake is usually included in epidemiologic studies to control for confounding between nutrient intakes and disease risk [44]. In relation to the risk of depression, several systematic reviews showed that fish or omega-3 fatty acid intake play a protective role against depression, particularly in women [45–47]. Thus, total energy intake and fish consumption measured using the food frequency questionnaire data from survey 5 were added in the models. Further adjustment for the history of depression (survey 1–3) was also included in models to examine the longitudinal association. Age and gender were not included in the models because all participants were women aged 25–30 at the current study baseline. The final analyses included 21,205 observations for a total of 4241 participants.

To test the robustness of our findings, a complete case analysis (listwise deletion) with the GEE model was performed, which omitted participants who had missing data on any of the variables used in the analysis. In addition, characteristics were compared with participants included in the analysis and excluded due to missing data on confounders. This analysis included 12,650 observations for a total of 2531 participants who have completed data in the five survey waves. All analyses were conducted with STATA software version 16 (IBM Corp, Armonk, New York, USA) using a two-sided 5% level of significance.

## Results

### Descriptive characteristics

The analysis included a total of 4241 participants representing 46.7% (4241/9081) of the total participants at baseline (Supplementary Fig. S1). These participants had complete data for daily intake of fruit and vegetable and the CESD-10 scores. Most of the characteristics of these participants differed from participants who were excluded in the analysis due to missing data on exposure and outcome of interest, except for age and history of chronic diseases (Table 1). Participants who were excluded from the analysis because of missing dietary information and the CESD-10 were more likely to have higher score in the CESD-10, be separated/divorced/widowed, have lower educational attainment, have difficulty in managing income, have a child, be a daily smoker, less likely to physically active, be a drinker, and be obese.

**Table 1:** Baseline comparison of characteristics of women who were in the analysis (*n* = 4241) and left out of the analysis due to missing exposure and outcome data (*n* = 4840)*

Characteristics at baseline (survey 3)	In analysis	Out of analysis*	*p* value^a^
	*n* = 4241	*n* = 4840	
Mean age (SD)	27.6 (1.45)	27.6 (1.46)	0.2
CESD-10	6.4 (4.98)	7.5 (5.49)	< 0.0001
Sociodemographic variables			
Area of residence			< 0.0001
Major cities	58.4%	53.8%	
Inner regional	25.3%	28.4%	
Outer regional/remote/very remote	16.3%	17.8%	
Marital status			< 0.0001
Married/ de facto	62.0%	60.8%	
Single	35.5%	34.4%	
Separated/divorced/widowed	2.5%	4.8%	
Education			< 0.0001
High school/less	22.4%	36.8%	
Trade/college	23.8%	27.3%	
University	53.8%	35.9%	
Ability to manage income			< 0.0001
No difficulty/not too bad	64.6%	52.1%	
Difficult sometimes	26.6%	33.2%	
Very difficult/impossible	8.8%	14.8%	
Having a child			< 0.0001
No	75.1%	61.6%	
Yes	24.9%	38.4%	
Health behaviours			
Smoking			< 0.0001
Never	62.1%	52.8%	
Ex-smoker	18.6%	18.4%	
Irregular smoker	4.9%	4.8%	
Weekly smoker	2.3%	2.9%	
Daily smoker	12.1%	21.2%	
Physical activity			< 0.0001
Nil/sedentary	7.3%	10.8%	
Low	39.2%	39.3%	
Moderate	23.9%	21.7%	
High	29.6%	28.2%	
Alcohol consumption			0.005
Non-drinker	7.0%	9.0%	
Rarely drinks	65.6%	57.1%	
Low-risk drinker	24.4%	29.7%	
Risky/high risk drinker	3.0%	4.2%	
BMI categories			< 0.0001
Acceptable weight	61.4%	55.7%	
Underweight	4.2%	4.8%	
Overweight	21.3%	22.8%	
Obese	13.1%	16.8%	
History of chronic diseases^b^			0.725
No	97.8%	97.7%	
Yes	2.2%	2.3%	
Fish consumption			0.034
Never	7.6%	8.6%	
< 30 g/day	58.4%	55.9%	
≥ 30 g/day	34.0%	35.5%	

*CESD* Center for Epidemiological Studies Depression, *SD* standard deviation

*Numbers may vary due to missing values

^a^*p* values from *t* test or chi-square test. All statistical tests were conducted using a two-sided 5% level of significance

^b^Chronic diseases in this study were heart disease, hypertension, diabetes

A total of 1281 participants reported having depression symptoms at least once across the surveys. Of these participants, 141 participants (3.3% of total) consistently showed symptoms of depression in the five survey waves (survey 3–8). A total of 2136 (50.4%) participants showed no depression symptoms in any surveys. The prevalence of depressive symptoms in the sample over time was moderately stable, ranging from 19.6 to 23.2%. There were 769 (18.1% of total) new cases of depression symptoms during the 15-year follow-up period from survey 3 (2003) to survey 8 (2018) after excluding 1607 who had a history of depression from survey 1 to survey 3.

Table 2 describes the proportions of fruit and vegetable consumption by women from each survey. The prevalence of women consuming the recommended daily intakes of fruit and vegetables fluctuated across surveys. In terms of fruit consumption, approximately 40% of women consumed at least two servings daily. The prevalence of women consuming the recommended servings of vegetables considerably low throughout the surveys. Overall, few women ate the recommended daily intake of fruit and vegetables, ranging from 3.4 to 11.6% across surveys.

**Table 2:** Distribution of fruit and vegetable consumption at each survey (*n* = 4241)

Survey	Survey 3 (%)	Survey 4* (%)	Survey 5 (%)	Survey 7 (%)	Survey 8 (%)
Fruit^a^					
≤ 1 serves/day	61.0	56.9	57.7	61.0	64
2–3 serves/day	35.5	39.7	40.3	37.5	34.1
≥ 4 serves/day	3.5	3.4	2.0	1.5	1.9
Vegetables^b^					
≤ 1 serves/day	5.2	26.1	2.8	12.4	11.6
2–4 serves/day	80.4	69.0	75.9	75.6	75.2
≥ 5 serves/day	14.3	4.9	21.3	12.0	13.2
Meet guidelines^c^	7.0	3.4	11.6	6.2	6.4

^a^A serve of fruit was equivalent to one medium piece or two small pieces of fruit, or a half cup of diced fruit, berries, or grapes

^b^A serve of vegetables was equivalent to half a cup (75 g) of cooked vegetables or a cup of salad vegetables

^c^In the Australian Dietary Guidelines, the minimum recommended daily intake of fruit and vegetables is two serves and five serves, respectively, for women aged 19–50

*For survey 4, the response categories for fruit and vegetable were: none, 1, 2–3, 4, and ≥ 5

### Cross-sectional analysis of fruit and vegetable consumption and depression symptoms

Table 3 presents the GEE analysis of cross-sectional associations between fruit and vegetable consumption and depression symptoms at each survey. Consuming fruit and vegetables were associated with lower odds of depressive symptoms after adjustment for energy intake, total fish consumption, and sociodemographic variables (Model 2). These associations remained strong even after health behaviours, BMI, and history of chronic diseases were included in the analysis (Model 3). The lowest odds of depression symptoms were observed among participants who consumed at least four servings of fruit each day with a 24% lower odds (OR 0.76; 95% CI 0.60, 0.96, *p* = 0.024) than participants who consumed one serve or less fruit. There was also a 21% lower odds among participants who consumed five or more servings of vegetables per day (OR 0.79; 95% CI 0.69, 0.91, *p* = 0.001) than participants who consumed one serve or less vegetable. Covariates associated with decreased odds of depression symptoms were having a child, low-risk drinking, and engaging with at least a low level of physical activity.

**Table 3:** Cross-sectional logistic regression models with GEE for associations between fruit and vegetable consumption and depression symptoms in the ALSWH 1973–78 cohort

	Model 1†		Model 2‡		Model 3§	
	OR	95% CI	OR	95% CI	OR	95% CI
Fruit						
≤ 1 serve/day	1.00	–	1.00	–	1.00	–
2–3 serves/day	**0.85**	**0.79–0.91**	**0.88**	**0.81–0.94**	**0.93**	**0.86–1.00**
≥ 4 serves/day	**0.69**	**0.55–0.86**	**0.71**	**0.56–0.89**	**0.76**	**0.60–0.96**
Vegetables						
≤ 1 serve/day	1.00	–	1.00	–	1.00	–
2–4 serves/day	**0.78**	**0.71–0.86**	**0.81**	**0.73–0.89**	**0.83**	**0.75–0.91**
≥ 5 serves/day	**0.71**	**0.63–0.81**	**0.75**	**0.66–0.86**	**0.79**	**0.69–0.91**

*GEE* generalised estimating equations, *OR* odds ratio, *CI* confidence interval

Bold indicates a significant association

^†^Adjusted for total energy intake and total fish consumption

^‡^Additionally adjusted for sociodemographic variables: area of residence, marital status, education, ability to manage on income, having a child

^§^Additionally adjusted for health behaviours: smoking status, physical activity, alcohol, BMI; and history of chronic disease

### Longitudinal analysis of fruit and vegetable consumption and depression symptoms

Similar results were observed in longitudinal analysis with GEE models (Table 4). Higher fruit consumption (≥ 4 servings) was consistently associated with lower odds of depression symptoms among women who consumed at least four servings of fruit (OR 0.75; 95% CI 0.57, 0.97; *p* = 0.031) than those consuming one serve or less of fruit in the fully adjusted model (Model 4). Also, significant associations were observed in vegetable consumption and lower odds of depression symptoms among women who consumed 2–4 servings of vegetables (OR 0.85; 95% CI 0.76, 0.95; *p* = 0.003) and five servings or more vegetables (OR 0.81; 95% CI 0.70, 0.94; *p* = 0.007) than those consuming one serve or less of vegetable. The longitudinal association between consuming 2–3 servings of fruit and lower odds of depression symptoms was consistent after controlling for sociodemographic variables (Model 3). However, the association was attenuated after health behaviours, BMI, and history of chronic disease were introduced in the analysis (Model 4). Several covariates (difficulty in managing income, being single, separated/divorced/widowed, a daily smoker, a risky/high-risk drinker, overweight, or obese) were significantly associated with increased odds of depression symptoms. On the other hand, having a child and engaging with at least a low level of physical activity were associated with decreased odds of depression symptoms.

**Table 4:** Longitudinal logistic regression models with GEE for associations between fruit and vegetable consumption and depression symptoms in the ALSWH 1973–78 cohort

	Model 1*		Model 2†		Model 3‡		Model 4§	
	OR	95% CI	OR	95% CI	OR	95% CI	OR	95% CI
*Fruit*								
≤ 1 serve/day	1.00	–	1.00	–	1.00	–	1.00	–
2**–**3 serves/day	**0.86**	**0.80–0.93**	**0.86**	**0.79–0.93**	**0.89**	**0.82–0.96**	0.94	0.87–1.02
≥ 4 serves/day	**0.69**	**0.53–0.89**	**0.68**	**0.53–0.88**	**0.69**	**0.53–0.90**	**0.75**	**0.57–0.97**
Vegetables								
≤ 1 serve/day	1.00	–	1.00	–	1.00	–	1.00	–
2**–**4 serves/day	**0.79**	**0.72–0.88**	**0.79**	**0.71–0.88**	**0.82**	**0.74–0.91**	**0.85**	**0.76–0.95**
≥ 5 serves/day	**0.72**	**0.63–0.84**	**0.72**	**0.62–0.83**	**0.76**	**0.66–0.88**	**0.81**	**0.70–0.94**

*GEE* generalised estimating equations, *OR* odds ratio, *CI* confidence interval

Bold indicates a significant association

^*^Adjusted for history of depression

^†^Additionally adjusted for total energy intake and total fish consumption

^‡^Additionally adjusted for sociodemographic variables: area of residence, marital status, education, ability to manage on income, having a child

^§^Additionally adjusted for health behaviours: smoking status, physical activity, alcohol, BMI, and history of chronic disease

### Complete case analysis

Complete case analysis included 2531 women in the analysis, excluding 1710 women because of missing confounders (Supplementary Table 2). Most missing data was due to BMI (8.8%) at the baseline, history of chronic diseases (6.8%) in survey 5, and physical activity status (6.5%) in survey 8. Women included in the complete case analysis were more likely to live in major cities, have higher educational attainment, be less likely to have a child, and consume lower fish consumption at the baseline. However, the mean of the CESD-10 scores did not differ between these groups.

The complete case analysis of higher consumption of fruit (≥ 4 servings) and vegetables (≥ 5 servings) did not alter the primary results in both cross-sectional and longitudinal after adjusting for covariates. Although consuming 2–3 servings of fruits lower the odds of depression symptoms in the cross-sectional and longitudinal analyses, the associations were weak. Similarly, the association between consuming 2–4 servings of vegetables and depression symptoms was attenuated in longitudinal analysis (Supplementary Table 3).

## Discussion

The present study provided evidence that higher intakes of fruit and vegetables (≥ 4 serving and ≥ 5 servings, respectively) were consistently associated with lower odds of depression symptoms over 15 years in Australian women in multiple imputation analysis and complete-case analysis. To our knowledge, this is the first study to provide evidence of the association between fruit and vegetable consumption and depression symptoms among young women over a 15-year follow-up.

We confirmed that a very low proportion of the sample population adheres to the recommended daily servings of fruit and vegetables, ranging from 3.4 to 11.6% across the surveys. This finding is common in population-based studies. For example, according to the Understanding Society study in the UK [48], only 13% of adults aged 15–29 and 20% of those aged 30–41 consumed the recommended daily intakes of fruit and vegetables. Furthermore, the rate of adherence to recommended amounts of vegetables was low among adults in three south Asian countries, with the highest rate was approximately 14% in Bangladesh, 7% in India, and 3% in Nepal based on data extracted from the World Health Survey [25].

Our cross-sectional findings are consistent with existing literature [49–52], which shows fruit and vegetable consumption (≥ 2 servings) was cross-sectionally associated with a lower risk of depression symptoms in young women. In alignment with our longitudinal findings, the evidence for sex differentials in the Whitehall II study showed that fruit and vegetables were independently associated with decreased odds of recurrent depression symptoms in women over 5 years [53]. Therefore, the authors suggested a long-term beneficial effect of adherence to the Alternative Healthy Eating Index (AHEI) to prevent depression symptoms due to specific nutrients provided by fruit and vegetables, such as high folate levels antioxidants. Similarly, a study conducted by Ocean et al. [48] found a dose–response effect between fruit and vegetable consumption and subjective well-being and highlighted the importance of increasing the quantity and frequency of fruit and vegetables consumed to increase mental well-being.

The findings of this study are generally in agreement with a recent systematic review and meta-analysis that found consumption of fruit and vegetable separately was associated with a lower risk of depression in adults based on the pooled relative risks in cross-sectional and cohort studies [54]. Likewise, a recent multi-national study concluded that inadequate fruit and vegetable consumption was associated with an increased likelihood of depression symptoms among adolescents in 25 low- and middle-income countries [55].

Our findings are somewhat different from the previous findings from the 1946–51 cohort of the ALSWH that found a reduction in the prevalence and incidence of depression symptoms with at least two servings of fruit, whereas the effects of vegetables were unclear [28]. We found a weak association between depression symptoms and women who consumed 2–3 servings of fruit (OR 0.94; 95% CI 0.87, 1.02; *p* = 0.152) than women consuming one serve or less fruit. Conversely, we found vegetable consumption at least two servings daily was significantly associated with lower odds of depression symptoms than participants consuming one serve or less vegetable in longitudinal analysis in the 1973–78 cohort of the ALSWH. Similar to our study, there was no observed association with fish consumption (Supplementary Table 4) [28].

Several observational studies have shown mixed results on associations between fruit and vegetable consumption and depression symptoms. Reasons for this discrepancy may have been because of the effects of factors, such as smoking, physical activity, alcohol, and BMI [16, 49, 56, 57]. For instance, a longitudinal study of US adolescents [49] showed a promising association between fruit consumption and depression with minimal adjustment for some covariates, but the association was attenuated after adjusting for BMI. Similarly, Winpenny et al. [56] reported that the association between fruit and vegetable consumption and depression was subsequently attenuated after controlling for behavioural covariates such as physical activity, smoking level, and alcohol consumption.

Further complicating matters, the associations between diet and depression symptoms are complex and plausibly bidirectional [57, 58]. A longitudinal community survey in Australia found evidence of reverse causality between dietary patterns and depression [59]. Likewise, findings from the Invecchiare in Chianti Study found depression symptoms were associated with 3-year decreases in vegetable consumption but not in fruit consumption [60]. The underlying mechanisms of reverse causality are not clear, but it has been suggested that emotional eating is one factor accounting for the relationship between depression symptoms and unhealthy food choices, including a lower intake of fruit and vegetables [61]. Emotional eating is considered a coping strategy in response to negative emotions, such as depression and anxiety, which may lead to changes in eating behaviour [61, 62].

### Plausible mechanisms

There are a number of concepts have been proposed to explain the mechanisms of action linking fruit and vegetable consumption with depression symptoms. The majority of findings are centred on the specific nutrients within fruit and vegetables, which influence psychological well-being [63]. Payne et al. [64] suggested that naturally occurring folate in foods, such as fruit and vegetables, may be more beneficial for preventing depression than dietary supplements and food fortification. The mechanisms proposed for their association have been linked to B vitamins because of their effects on single-carbon metabolism and their role in the synthesis of neurotransmitters, including serotonin, other monoamine neurotransmitters, and catecholamines [65, 66]. Furthermore, the critical role of B vitamins in brain function is demonstrated by a number of neuropsychiatric symptoms commonly associated with deficiencies in any of the B vitamins, including folate and vitamin B_12_ [66, 67]. A recent study found that the relationship between dietary patterns and depression is mediated by serum levels of folate and vitamin B_12_ [68].

High concentrations of antioxidants in fruits and vegetables also present a promising link to psychological well-being. Depleted non-enzymatic antioxidants are considered to be the causative factors for oxidative stress, which may exhibit the development of the major depressive disorder in the long term [69]. A recent study compiled a list of fresh fruits and vegetables which have the potential to prevent chronic human diseases [70]. Among fruits, berries have high antioxidants and phytochemicals such as flavonoids, tannins, and lignans [70]. In alignment with this finding, two cross-sectional studies found that berries have been inversely associated with depressive symptoms [71, 72]. With regard to vegetables, broccoli, carrot, tomato, pea, and sweet pepper are vegetables with rich sources of phytochemical α-carotene and antioxidant β-carotene [70].

### Strengths and limitations

The strengths of our study include the use of data from a large population-based prospective cohort with repeated measurements of fruit and vegetable consumption, depression symptoms, and other health-related behaviours over 15 years. In addition, the assessment of depression symptoms utilised a validated and tested CESD-10 scale. We also adjusted for key confounders such as sociodemographic, lifestyle factors, body mass index, history of chronic disease, and other aspects of diet (total energy intake and fish consumption), which may reduce unmeasured residual confounding. Multiple imputations by chained equations was performed to improve the statistical power by imputing missing values, which is common in large datasets. Additionally, a complete case analysis was also presented to compare the results.

Our findings should also be interpreted in light of several limitations. First, almost half of the women had missing data on fruit and vegetable consumption and the CESD-10, which may introduce selection bias. In addition, we omitted the outcome variable from the imputation procedure. Some criticisms of the usage of MICE are that it does not have the same theoretical justification as other imputation approaches, and the imputation process unacceptably slow when the dataset contains many variables (e.g. several nominal categorical variables imputed by multinominal logistic regression) [73]. Furthermore, due to the exclusion of Survey 6 from the analysis, the direction of the odds of depression symptoms may bias the results towards the null.

The assessment method using self-report of both dietary intake and depression symptoms may be subject to reporting bias. In addition, inconsistency in short questions and answer choices used to measure the daily serving of fruit and vegetables over time may be prone to measurement error and confounding.

Although the history of depression prior to and at the baseline was adjusted for in the longitudinal analysis, the possibility of reverse causation may exist due to its observational nature. Finally, unmeasured residual confounding (e.g., psychosocial factors) that are associated with depression symptoms may have introduced bias. Depression symptom in the current study is not reflective of clinical depression. With regard to generalisability, it is difficult to generalise the results to men or other age groups. Further studies in these groups are highly recommended to confirm our findings.

## Conclusion

Our findings demonstrate that higher consumption of fruit and vegetables was associated with a lower risk of depression symptoms over a 15-year in young women from a population-based prospective study in Australia. Our results also support the evidence that fruit and vegetable consumption could be an important predictor of depression symptoms. To increase confidence in study findings, replication of the results in other populations is necessary. Improving dietary patterns to include more fruit and vegetables may be an essential component of strategies to prevent depression.

## Supplementary Information

Below is the link to the electronic supplementary material.

Supplementary file1 (DOCX 48 KB)

## Data Availability

Data are available from the Research Centre for Gender, Health, and Ageing (RCGHA) at the University of Newcastle and School of Public Health at the University of Queensland on request.
