# Supplementary material for: Fruit and vegetable consumption and depression symptoms in young women: results from 1973 to 1978 cohort of the Australian Longitudinal Study on Women’s Health
Source: Eur J Nutr. 2022 Jul 21;61(8):4167–78. doi: 10.1007/s00394-022-02926-8 (PMC9596510; doi:10.1007/s00394-022-02926-8)
Supplement: Supplementary file 1 — Supplementary file1 (DOCX 48 KB) [file 394_2022_2926_MOESM1_ESM.docx]

Women who completed survey 3 in 2003 (n = 9,081)

Missing data on fruit and vegetable consumption and/or CESD-10 scores at survey 3

n = 163

Sample for analysis of the association between fruit and vegetable consumption and depression symptoms (n = 4,241)

Women who followed survey 4 in 2006 (n = 8,918)

Missing data on fruit and vegetable consumption and/or CESD-10 scores at survey 4

n = 1,397

Women who followed survey 5 in 2009 (n = 7,521)

Women who followed survey 7 in 2015 (n = 6,196)

Missing data on fruit and vegetable consumption and/or CESD-10 scores at survey 7

n = 1,365

Missing data on fruit and vegetable consumption and/or CESD-10 scores at survey 5

n = 1,325

Women who followed survey 8 in 2018 (n = 4,831)

Missing data on fruit and vegetable consumption and/or CESD-10 scores at survey 8

n = 590

**Supplemental Fig. 1.** Flowchart of the participants included in the current analysis.

**Supplementary table 1***.* The short questions to assess the daily intakes of fruit and vegetable.

|  | Fruit | | Vegetable | |
| --- | --- | --- | --- | --- |
|  | Question | Answer choices | Question | Answer choices |
| Survey 3 & 5 | How many pieces of fresh fruit do you usually eat per day? | - I don’t eat fruit - Less than 1 piece of fruit per day - 1 piece of fruit per day - 2 pieces of fruit per day - 3 pieces of fruit per day - 4 or more pieces of fruit per day | How many different vegetables do you usually eat per day? | - Less than 1 vegetable per day - 1 vegetable per day - 2 vegetables per day - 3 vegetables per day - 4 vegetables per day - 5 vegetables per day - 6 or more vegetables per day |
| Survey 4 | How many serves of fruit do you usually eat each day? | - None - 1 serve - 2-3 serves - 4 serves - 5 serves or more | How many serves of vegetables do you usually eat each day? | - None - 1 serve - 2-3 serves - 4 serves - 5 serves or more |
| Survey 7 & 8 | How many pieces of fresh fruit do you usually eat per day? | - I don’t eat fruit - Less than 1 piece of fruit per day - 1 piece of fruit per day - 2 pieces of fruit per day - 3 pieces of fruit per day - 4 or more pieces of fruit per day | How many serves of vegetables do you usually eat each day? | - None - Less than one serve - 1 serve - 2 serves - 3 serves - 4 serves - 5 serves or more |

**Supplementary table 2.** Comparison of characteristics of women who were in the study and left out of the analysis owing to missing confounding data.

| Characteristics at baseline (survey 3) | Complete-case analysis | Out of analysis^a^ | p-value^b^ |
| --- | --- | --- | --- |
|  | n = 2,531 | n = 1,710 |  |
| Mean age (SD) | 27.6 (1.46) | 27.6 (1.44) | 0.734 |
| CESD-10 | 6.47 (5.01) | 6.40 (4.94) | 0.651 |
| Socio-demographic variables |  |  |  |
| *Area of residence* |  |  | 0.001 |
| Major cities | 60.65% | 55.06% |  |
| Inner regional | 23.63% | 27.76% |  |
| Outer regional/remote/very remote | 15.73% | 17.18% |  |
| *Marital status* |  |  | 0.003 |
| Married/ de facto | 59.94% | 65.08% |  |
| Never married | 37.46% | 32.51% |  |
| Separated/divorced/widowed | 2.61% | 2.41% |  |
| *Education* |  |  | 0.001 |
| High school/less | 21.17% | 24.32% |  |
| Trade/college | 22.68% | 25.33% |  |
| University | 56.15% | 50.36% |  |
| *Ability to manage on income* |  |  | 0.493 |
| No difficulty/not too bad | 64.99% | 63.93% |  |
| Difficult sometimes | 26.00% | 27.57% |  |
| Very difficult/impossible | 9.01% | 8.50% |  |
| *Having a child* |  |  | <0.0001 |
| No | 77.12% | 72.05% |  |
| Yes | 22.88% | 27.95% |  |
| Health behaviours | | | |
| *Smoking* |  |  | 0.640 |
| Never | 62.70% | 61.13% |  |
| Ex-smoker | 18.02% | 19.50% |  |
| Irregular smoker | 5.14% | 4.64% |  |
| Weekly smoker | 2.37% | 2.29% |  |
| Daily smoker | 11.77% | 12.45% |  |
| *Physical activity* |  |  | 0.245 |
| Nil/sedentary | 6.95% | 7.83% |  |
| Low | 38.31% | 40.55% |  |
| Moderate | 24.42% | 23.09% |  |
| High | 30.30% | 28.53% |  |
| *Alcohol consumption* |  |  | 0.245 |
| Non-drinker | 6.12% | 8.41% |  |
| Rarely drinks | 24.30% | 24.47% |  |
| Low risk drinker | 66.69% | 64.00% |  |
| Risky/high risk drinker | 2.88% | 3.12% |  |

**Supplementary table 2.** Continued

| Characteristics at baseline (survey 3) | Complete-case analysis | Out of analysis^a^ | p-value^b^ |
| --- | --- | --- | --- |
|  | n = 2,531 | n = 1,710 |  |
| Health behaviours | | | |
| *BMI categories* |  |  | 0.397 |
| Acceptable weight | 61.56% | 61.06% |  |
| Underweight | 3.87% | 4.78% |  |
| Overweight | 21.77% | 20.48% |  |
| Obese | 12.80% | 13.68% |  |
| History of chronic disease^c^ |  |  | 0.878 |
| No | 97.75% | 97.82% |  |
| Yes | 2.25% | 2.18% |  |
| Fish intake |  |  | 0.007 |
| Never | 8.18% | 6.73% |  |
| <30g/day | 59.54% | 56.67% |  |
| >30g/day | 32.28% | 36.61% |  |

CESD, Center for Epidemiological Studies Depression; SD, standard deviation; BMI, body mass index

^a^ Numbers may vary due to missing baseline values.

^b^ p-values from t-test or chi-square test. All statistical tests were conducted using a two-sided 5% level of significance.

^c^ Chronic diseases in this study were heart disease, hypertension, diabetes.

**Supplementary Table 3**. Comparison logistic regression models with GEE for associations between fruit and vegetable consumption and depression symptoms on the ALSWH 1973-78 cohort between multiple imputation analysis (n= 4,241) and complete-case analysis (n= 2,531).

|  | Imputation† | | Complete-case† | |
| --- | --- | --- | --- | --- |
| Cross-sectional | OR | 95% CI | OR | 95% CI |
| *Fruit* |  |  |  |  |
| < 1 serve/day | 1.00 | - | 1.00 | - |
| 2-3 serves/day | **0.93** | **0.86-1.00** | 0.96 | 0.87-1.06 |
| > 4 serves/day | **0.76** | **0.60-0.96** | **0.61** | **0.44-0.86** |
| *Vegetables* |  |  |  |  |
| < 1 serve/day | **1.00** | **-** | 1.00 | - |
| 2-4 serves/day | **0.83** | **0.75-0.91** | **0.85** | **0.75-0.97** |
| > 5 serves/day | **0.79** | **0.69-0.91** | **0.73** | **0.61-0.87** |
| Longitudinal |  |  |  |  |
| *Fruit* |  |  |  |  |
| < 1 serve/day | 1.00 | - | 1.00 | - |
| 2-3 serves/day | 0.94 | 0.87-1.02 | 0.99 | 0.89-1.10 |
| > 4 serves/day | **0.75** | **0.57-0.97** | **0.62** | **0.43-0.89** |
| *Vegetables* |  |  |  |  |
| < 1 serve/day | 1.00 | - | 1.00 | - |
| 2-4 serves/day | **0.85** | **0.76-0.95** | 0.88 | 0.77-1.02 |
| > 5 serves/day | **0.81** | **0.70-0.94** | **0.74** | **0.61-0.90** |
| Total observations | 21,205 | | 12,655 | |

GEE, generalised estimating equations; OR, odds ratio; CI, confidence interval.

Bold indicates a significant association.

† Fully adjusted for all confounders.

**Supplementary Table 4.** Fully adjusted report of longitudinal logistic regression models with GEE for associations between fruit and vegetable consumption and depression symptoms in the ALSWH 1973–78 cohort.

|  | Odd Ratio (95% CI) | P-value |
| --- | --- | --- |
| *Fruit* |  |  |
| < 1 serve/day | 1.00 | - |
| 2-3 pieces/day | 0.94 (0.87-1.02) | 0.164 |
| >= 4 pieces/day | **0.75 (0.57-0.97)** | **0.031** |
| *Vegetables* |  |  |
| < 1 serve/day | 1.00 | - |
| 2-4 serves/day | **0.85 (0.76-0.95)** | **0.003** |
| >=5 serves/day | **0.81 (0.70-0.94)** | **0.007** |
| *History of depression* | **6.27 (5.69-6.92)** | **<0.001** |
| *Total energy intake* | 1.00 (1.00-1.00) | 0.086 |
| *Fish consumption* |  |  |
| None | 1.00 | - |
| <30 g/day | 0.91 (0.76-1.09) | 0.316 |
| >=30 g/day | 0.89 (0.74-1.07) | 0.227 |
| *Area* |  |  |
| Major cities | 1.00 | - |
| Inner regional | 1.05 (0.95-1.16) | 0.354 |
| Outer regional/remote/very remote | 1.04 (0.92-1.18) | 0.526 |
| *Income* |  |  |
| No difficulty/not too bad | 1.00 | - |
| Difficult sometimes | **1.34 (1.23-1.46)** | **<0.001** |
| Very difficult/impossible | **2.37 (2.12-2.66)** | **<0.001** |
| *Education* |  |  |
| High school or less | 1.00 | - |
| Trade/College | 1.04 (0.90-1.21) | 0.597 |
| University | 0.99 (0.86-1.14) | 0.898 |
| *Marital status* |  |  |
| Married/De facto | 1.00 | - |
| Single | **1.36 (1.22-1.51)** | **<0.001** |
| Seperated/ Divorced/ Widowed | **1.68 (1.45-1.95)** | **<0.001** |
| *Have a child* | **0.89 (0.82-0.97)** | **0.009** |
| *Smoking* |  |  |
| Never smoker | 1.00 | - |
| Ex-smoker | 1.01 (0.91-1.12) | 0.862 |
| Irregular smoker | 1.20 (0.98-1.48) | 0.085 |
| Weekly smoker | 1.08 (0.80-1.46) | 0.609 |
| Daily smoker | **1.17 (1.01-1.35)** | **0.032** |

**Supplementary Table 4.** *Continued*

|  | Odd Ratio (95% CI) | P-value |
| --- | --- | --- |
| *Alcohol consumption* |  |  |
| Non-drinker | 1.00 | - |
| Low risk drinker | 0.88 (0.76-1.01) | 0.063 |
| Rarely drinks | 0.96 (0.83-1.11) | 0.570 |
| Risky/High Risk drinker | **1.35 (1.10-1.67)** | **0.005** |
| *Physical activity* |  |  |
| Nil/Sedentary | 1.00 | - |
| Low | **0.76 (0.67-0.85)** | **<0.001** |
| Moderate | **0.68 (0.60-0.78)** | **<0.001** |
| High | **0.57 (0.50-0.65)** | **<0.001** |
| *BMI* |  |  |
| Acceptable weight, 18.5 <= BMI < 25 | 1.00 | - |
| Underweight, BMI < 18.5 | 1.06 (0.84-1.36) | 0.615 |
| Overweight, 25 <= BMI < 30 | **1.12 (1.02-1.24)** | **0.021** |
| Obese, 30 <= BMI | **1.36 (1.22-1.52)** | **<0.001** |
| *History of chronic disease* | 1.15 (0.96-1.38) | 0.121 |

GEE, generalised estimating equations; OR, odds ratio; CI, confidence interval; BMI, body mass index.

Bold indicates a significant association.
